# A High-Protein, Low Glycemic Index Diet Suppresses Hunger but Not Weight Regain After Weight Loss: Results From a Large, 3-Years Randomized Trial (PREVIEW)

**DOI:** 10.3389/fnut.2021.685648

**Published:** 2021-06-01

**Authors:** Ruixin Zhu, Mikael Fogelholm, Thomas M. Larsen, Sally D. Poppitt, Marta P. Silvestre, Pia S. Vestentoft, Elli Jalo, Santiago Navas-Carretero, Maija Huttunen-Lenz, Moira A. Taylor, Gareth Stratton, Nils Swindell, Niina E. Kaartinen, Tony Lam, Teodora Handjieva-Darlenska, Svetoslav Handjiev, Wolfgang Schlicht, J. Alfredo Martinez, Radhika V. Seimon, Amanda Sainsbury, Ian A. Macdonald, Margriet S. Westerterp-Plantenga, Jennie Brand-Miller, Anne Raben

**Affiliations:** ^1^Department of Nutrition, Exercise and Sports, Faculty of Science, University of Copenhagen, Copenhagen, Denmark; ^2^Department of Food and Nutrition, University of Helsinki, Helsinki, Finland; ^3^Human Nutrition Unit, School of Biological Sciences, Department of Medicine, University of Auckland, Auckland, New Zealand; ^4^Center for Health Technology Services Research, NOVA Medical School, Universidade Nova de Lisboa, Lisbon, Portugal; ^5^Centre for Nutrition Research, University of Navarra, Pamplona, Spain; ^6^Centro de Investigacion Biomedica en Red Area de Fisiologia de la Obesidad y la Nutricion (CIBEROBN), Madrid, Spain; ^7^IdisNA Instituto for Health Research, Pamplona, Spain; ^8^Institute for Nursing Science, University of Education Schwäbisch Gmünd, Schwäbisch Gmünd, Germany; ^9^Division of Physiology, Pharmacology and Neuroscience, School of Life Sciences, Queen's Medical Centre, Nottingham, United Kingdom; ^10^National Institute for Health Research (NIHR) Nottingham Biomedical Research Centre, Nottingham, United Kingdom; ^11^Applied Sports, Technology, Exercise and Medicine (A-STEM) Research Centre, Swansea University, Swansea, United Kingdom; ^12^Department of Public Health and Welfare, Finnish Institute for Health and Welfare, Helsinki, Finland; ^13^NetUnion sarl, Lausanne, Switzerland; ^14^Department of Pharmacology and Toxicology, Medical University of Sofia, Sofia, Bulgaria; ^15^Exercise and Health Sciences, University of Stuttgart, Stuttgart, Germany; ^16^Department of Nutrition and Physiology, University of Navarra, Pamplona, Spain; ^17^Precision Nutrition and Cardiometabolic Health Program, IMDEA (Madrid Institute for Advanced Studies)-Food Institute, CEI UAM + CSIC (Campus de Excelencia Internacional, Universidad Autónoma de Madrid + Consejo Superior de Investigaciones Científicas), Madrid, Spain; ^18^The Boden Collaboration for Obesity, Nutrition, Exercise, and Eating Disorders, Faculty of Medicine and Health, Charles Perkins Centre, The University of Sydney, Camperdown, NSW, Australia; ^19^School of Human Sciences (Exercise and Sports Science), Faculty of Science, The University of Western Australia, Crawley, WA, Australia; ^20^Division of Physiology, Pharmacology and Neuroscience, School of Life Sciences, Queen's Medical Centre, MRC/ARUK Centre for Musculoskeletal Ageing Research, ARUK Centre for Sport, Exercise and Osteoarthritis, National Institute for Health Research (NIHR) Nottingham Biomedical Research Centre, Nottingham, United Kingdom; ^21^Department of Nutrition and Movement Sciences, NUTRIM, School of Nutrition and Translational Research in Metabolism, Maastricht University, Maastricht, Netherlands; ^22^School of Life and Environmental Sciences and Charles Perkins Centre, The University of Sydney, Sydney, NSW, Australia; ^23^Steno Diabetes Center Copenhagen, Gentofte, Denmark

**Keywords:** obesity, pre-diabetes, satiety, desire to eat, low-energy diet, weight-loss maintenance

## Abstract

**Background:** Previous studies have shown an increase in hunger during weight-loss maintenance (WLM) after diet-induced weight loss. Whether a combination of a higher protein, lower glycemic index (GI) diet and physical activity (PA) can counteract this change remains unclear.

**Aim:** To compare the long-term effects of two diets [high protein (HP)-low GI vs. moderate protein (MP)-moderate GI] and two PA programs [high intensity (HI) vs. moderate intensity (MI)] on subjective appetite sensations during WLM after ≥8% weight loss (WL).

**Methods:** Data derived from the 3-years PREVIEW randomized intervention study. An 8-weeks WL phase using a low-energy diet was followed by a 148-weeks randomized WLM phase. For the WLM phase, participants were assigned to one of the four groups: HP-MI, HP-HI, MP-MI, and MP-HI. Available data from 2,223 participants with overweight or obesity (68% women; BMI ≥ 25 kg/m^2^). Appetite sensations including satiety, hunger, desire to eat, and desire to eat something sweet during the two phases (at 0, 8 weeks and 26, 52, 104, and 156 weeks) were assessed based on the recall of feelings during the previous week using visual analogue scales. Differences in changes in appetite sensations from baseline between the groups were determined using linear mixed models with repeated measures.

**Results:** There was no significant diet × PA interaction. From 52 weeks onwards, decreases in hunger were significantly greater in HP-low GI than MP-moderate GI (*P*_time × *diet*_ = 0.018, *P*_dietgroup_ = 0.021). Although there was no difference in weight regain between the diet groups (*P*_time × *diet*_ = 0.630), hunger and satiety ratings correlated with changes in body weight at most timepoints. There were no significant differences in appetite sensations between the two PA groups. Decreases in hunger ratings were greater at 52 and 104 weeks in HP-HI vs. MP-HI, and greater at 104 and 156 weeks in HP-HI vs. MP-MI.

**Conclusions:** This is the first long-term, large-scale randomized intervention to report that a HP-low GI diet was superior in preventing an increase in hunger, but not weight regain, during 3-years WLM compared with a MP-moderate GI diet. Similarly, HP-HI outperformed MP-HI in suppressing hunger. The role of exercise intensity requires further investigation.

**Clinical Trial Registration:** www.ClinicalTrials.gov, identifier: NCT01777893.

## Introduction

Obesity prevalence worldwide is rapidly increasing. Obesity considerably increases the risk of multiple serious diseases, and places a huge public health and economic burden on individuals and governments (1). In obesity management, maintaining weight loss (WL) and preventing weight regain are much more challenging than simply losing weight (2). Lifestyle interventions via dietary change and/or increased physical activity (PA) may aid WL in the short-term, whereas many individuals regain weight in the long-term (3, 4). It has been reported that no more than 20% of individuals who managed to lose weight can maintain 10% WL over 1 year (5).

Weight regain may be partly attributed to physiological adaptations, for example, a decrease in energy expenditure or an increase in drive to eat (1, 6–9). For instance, diet-induced WL has been shown to be linked with increased feelings of hunger and ghrelin secretion, an orexigenic hormone, and decreased satiety-related hormones (10–12). An increase in hunger has also been found in two studies during long-term weight-loss maintenance (WLM) achieved by a combination of energy-restricted diets and exercise (12, 13). These two studies, however, did not address dietary macronutrient composition, also known to affect appetite control (14–16). A systematic review has suggested that higher protein intake might affect appetite by increasing fullness or satiety in individuals with overweight or obesity (17), whereas the effect of carbohydrate and glycemic index (GI) on appetite remains highly controversial (18–21). In addition PA can help prevent obesity (22) and alter appetite control by promoting an energy deficit and inducing changes in physiological response (23, 24). The intensity of PA may also play a role in appetite regulation, but previous studies testing this hypothesis were all short-term (25–28).

To the best of our knowledge, there is no long-term study with repeated measures design addressing the effect of dietary macronutrient composition, GI, and PA intensity together on changes in appetite sensations. Therefore, the main objective of the present analysis was to compare the long-term effects of two diets [high protein (HP)-low GI vs. moderate protein (MP)-moderate GI diet] or two PA programs (moderate vs. high intensity PA) on subjective appetite sensations after ≥8% weight loss over 8 weeks in adults with overweight and pre-diabetes in the PREVIEW study. The second aim was to determine the effects of four diet-PA interventions. Our primary hypothesis was that the HP-low GI diets would be superior in suppressing appetite compared with MP-moderate GI diets and high intensity PA (HI) would be superior compared with moderate intensity PA (MI).

## Materials and Methods

### Study Design

This secondary analysis was based on the data from PREVIEW, a 3-years multi-center, 2 × 2 factorial randomized controlled trial (RCT) conducted at eight intervention centers in Denmark, Finland, the UK, the Netherlands, Spain, Bulgaria, Australia, and New Zealand. Detailed information has been published in the PREVIEW protocol and main papers (29–31). Briefly, the aim of the PREVIEW study was to explore the effects and interactions of two diets and two PA programs on the prevention of type 2 diabetes. The PREVIEW study comprised two intervention phases. Phase 1 was an 8-weeks WL phase. During this period, participants were provided with a commercial liquid low-energy diet (LED, 3.4 MJ/d). Those who lost≥8% of their initial body weight (BW) were allowed to enter phase 2, a 148-weeks WLM intervention period. The four diet-PA intervention groups were: (1) HP-low GI, MI (HP-MI); (2) HP-moderate GI, HI (HP-HI); (3) MP-moderate GI, MI (MP-MI); and (4) MP-moderate GI, HI (MP-HI). The HP-low GI diet targeted 25 E% protein and 45 E% carbohydrate with GI <50; the MP-moderate GI diet targeted 15 E% protein and 55 E% carbohydrate with GI > 56. The MI group aimed to achieve moderate intensity PA: 3–5.9 metabolic equivalents of task (MET) for 150 min/week (=450 MET·min/week); the HI group aimed to achieve high intensity PA: ≥6 METs for 75 min/weeks (=450 MET·min/weeks) as recommended (32). During WLM, participants were encouraged to maintain the WL they had achieved; and further WL was permitted. Participants were guided on the proportions and types of foods that they should consume from different food groups, to achieve the macronutrient content and GI required; whilst being encouraged to select portions sizes for themselves that they felt were appropriate for them. In addition, they were provided with examples of eating plans, food-exchange lists, and cooking books to help them meet the requirements of the intervention diets. A healthy food pattern was emphasized in all groups. The participants were also given instructions and small booklets on how to achieve the PA targets. Underpinning the program was the PREVIEW Behavior Modification Tool (PREMIT), which assisted participants in developing the necessary behavior change skills in order to adopt their new diet and PA behaviors and to ensure the changes became embedded as habits (33). PREMIT was incorporated in the 17 group visits (8–12 participants) throughout the study.

A total of seven clinical investigation days (CIDs) were undertaken at 0, 8, 26, 52, 78, 104, and 156 weeks to measure outcomes including anthropometry and questionnaires regarding appetite sensations. At each CID, participants arrived in the morning in a fasted state. The diet compliance was assessed by 4-days food records and 24-h urine N analysis; the PA compliance was examined by 7-days accelerometry.

The dietary compliance has been reported in the PREVIEW main results paper (31). In brief, from the 4-days food records, the HP-MI and HP-HI groups had higher protein and fat intake and lower carbohydrate intake and GI than the MP-MI and MP-HI groups during WLM (HP-MI: protein~22 E%, carbohydrate~38 E%, fat~35%, GI~51; HP-HI: protein~22 E%, carbohydrate~37 E%, fat~35%, GI~51; MP-MI: protein~19 E%, carbohydrate~42 E%, fat~34%, GI~55; MP-HI: protein~19 E%, carbohydrate~43 E%, fat~33%, GI~56). There were no significant differences between the four intervention groups in dietary fiber and energy intake at each CID, with the exception of a higher energy intake in the HP-MI compared with the MP-HI group at 52 weeks. There were no significant differences between the four intervention groups in total, vigorous, and moderate-to-vigorous PA or sedentary time, which means that PA recommendations were not completely followed, although PA increased in all four groups.

The PREVIEW study was approved by the Human Ethics Committees at each intervention center [Denmark: The Research Ethics Committees of the Capital Region; Finland: Coordinating Ethical Committee of HUS (Helsinki and Uusimaa Hospital District); the UK: UK National Research Ethics Service (NRES) and East Midlands (Leicester) Ethics Committee; the Netherlands: Medical Ethics Committee of the Maastricht University Medical Centre; Spain: Research Ethics Committee of the University of Navarra; Bulgaria: Commission on Ethics in Scientific Research with the Medical University-Sofia (KENIMUS); Australia: The University of Sydney, Human Research Ethics Committee (HREC); and New Zealand: Health and Disability Ethics Committees (HDEC)]. The study was conducted in accordance with the Declaration of Helsinki and its later amendments and was registered at ClinicalTrials.gov as NCT01777893.

### Participants

The inclusion and exclusion criteria of the PREVIEW study have been described elsewhere (29). In brief, the inclusion criteria were adults aged 25–70 years and with BMI≥25 kg/m^2^ and pre-diabetes. Pre-diabetes was defined in accordance with American Diabetes Association criteria (34): (1) fasting plasma glucose (FPG) of 5.6–6.9 mmol/L and/or (2) plasma glucose of 7.8–11.0 mmol/L at 2 h in an oral glucose tolerance test (OGTT) and FPG <7.0 mmol/L. The main exclusion criterion was individuals with pre-existing diabetes.

Adult participants were recruited from June 2013 to April 2015 via multiple methods. Participants were selected via a pre-screening tool or an interview including questions relating to the Finnish Diabetes Risk Score (35) and relevant to inclusion and exclusion criteria. Participants who met the above-mentioned criteria provided written informed consent and were invited to the laboratories for a final physical examination. During final screening, BW, height, and resting blood pressure were assessed and an OGTT with 75 g glucose was conducted. Eligible participants were enrolled, randomized to one of the four intervention groups, and started the WL phase. The randomization was stratified based on sex and age groups (≤ 45, 46–54, and >54 years). In this secondary analysis, data are from all available participants who commenced the WL phase.

### Assessment of Subjective Appetite Sensations

Overall subjective appetite sensations were assessed at the clinical site using electronic questionnaires with separate visual analogue scales (VAS), validated previously (36–38). In the morning during the OGTT, participants were asked to consider their overall feelings of satiety, hunger, desire to eat, and desire to eat something sweet during the previous week. Participants were asked to answer the following questions: How satisfied have you felt? How hungry have you felt? Have you felt a desire to eat? Have you felt like eating something sweet? Responses to the four different questions were marked on the VAS. There were four 100-mm horizontal lines anchored with extreme appetite sensations [left: very little (0)–right: very much (100)] on both ends of each line in the questionnaire. Further analyses were based on changes in appetite sensation ratings from 0 to 8, 26, 52, 104, and 156 weeks.

### Measurement of Body Weight

BW has been previously reported in the PREVIEW main paper (31) but to aid comprehension, this measure has also been reported in the present study. BW was measured when participants were in >10-h fasting state and lightly clad. Further analyses were based on changes in BW from 0 to 8, 26, 52, 78, 104, and 156 weeks.

### Statistical Analysis

The sample size was calculated based on the primary outcome in the PREVIEW study (29). For descriptive statistics, data are presented as means±SD, median (25th, 75th percentiles) or the number of participants (%) where appropriate.

Differences in weight and in absolute scores for each appetite sensation from baseline to the end of WL (0–8 weeks) and from baseline to the end of WLM (0–156 weeks) were assessed using Mann-Whitney *U* non-parametric tests.

For differences in appetite and weight outcomes between the two diet groups or two PA groups, a 3-way interaction of diet × PA × time was tested using linear mixed models. In addition, a 2-way interaction of diet × time and PA × time was also performed to assess the single effects of diet or PA groups over time. If the interaction was significant, *post hoc* pairwise comparisons were conducted at each time point.

For differences in appetite and weight outcomes between the four diet-PA arms, a 2-way interaction of diet-PA group × time was tested using linear mixed models with repeated measures. If the interaction was significant, *post hoc* multiple comparisons with Bonferroni correction were conducted at each time point.

The models for the four groups or two diets or two PA groups were adjusted for fixed factors including age (continuous), sex (categorical), ethnicity (categorical: Caucasian, Asian, Black, Arabic, Hispanic, or other), baseline outcomes (continuous: 0 weeks), and time (categorical) adjusted for random factors including participant-ID and intervention center (categorical). As ignoring stratification factors in the analysis may cause larger *P*-values and wider confidence intervals, age, sex, and other balancing factors including ethnicity and baseline outcomes were included in the models (39). The above-mentioned analysis was repeated by additionally adjusting for time-varying changes in energy intake from baseline. The energy from LED (3.4 MJ/d) was considered to be energy intake at 8 weeks. Energy intake from 26 to 156 weeks was determined by 4-days food records. The interaction of group × sex or group × age (≤ 45, 46–54, and >54 years) was also investigated. Both main effects and interactions were reported.

The correlation between changes in appetite sensation ratings and change in body weight from 0 to 26, 52, 78, 104, and 156 weeks were evaluated by merging participants from the four intervention groups into one group. Spearman correlation coefficients were used.

*P* < 0.05 (two-tailed) were considered statistically significant. All statistical analyses were performed based on available data from participants who entered the WL phase and were conducted using Statistical Package for the Social Sciences 26.0 (SPSS, Chicago, IL).

## Results

**Figure 1:**
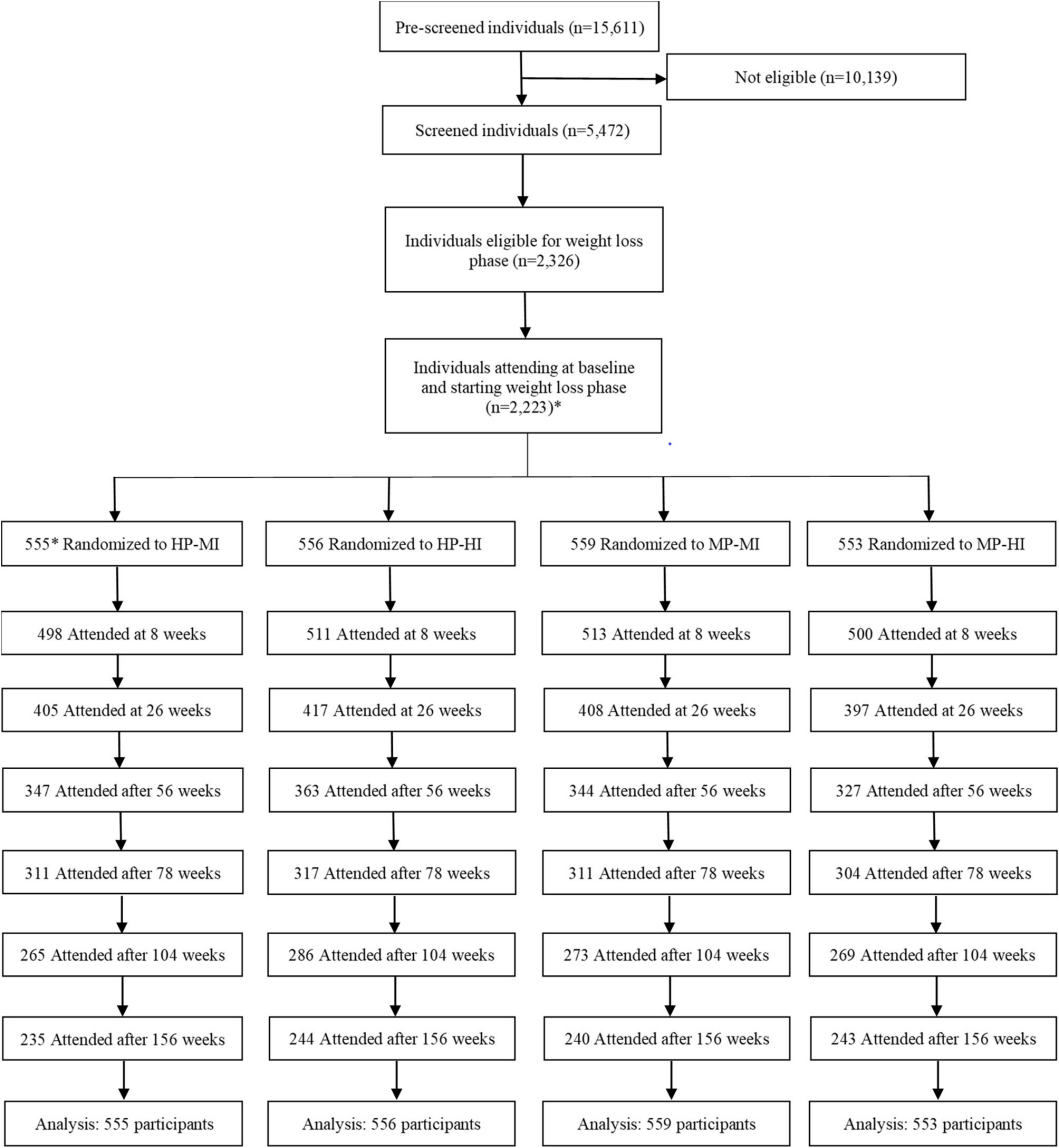
Trial flow diagram. *A total of 2,224 individuals participated and 556 were randomized to the HP-MI group, but one of them withdrew consent and requested data deletion. HP-MI, high protein-low glycemic index diet, moderate intensity physical activity; HP-HI, high protein-low glycemic index diet, high intensity physical activity; MP-MI, moderate protein-moderate glycemic index diet, moderate intensity physical activity; MP-HI, moderate protein-moderate glycemic index diet, high intensity physical activity.

A total of 2,326 individuals were eligible for the trial and 2,224 were enrolled, randomized into the four intervention groups, and started the WL phase (68% women) (Figure 1). Data from 2,223 participants were included in the analysis. Baseline characteristics were similar in the four intervention groups (Table 1). In total, 962 participants (52%) completed the 3-years study and the attrition rate during 3 years was similar in the four intervention groups.

**Table 1:** Baseline (0 weeks) socio-demographics, anthropometric, and appetite sensation characteristics of the participants in the four intervention groups.

**Characteristic**	**HP-MI**	**HP-HI**	**MP-MI**	**MP-HI**
N	555*	556	559	553
Female, n (%)	371 (66.8)	379 (68.2)	379 (67.8)	374 (67.6)
Age (year)	55 (43, 61)	55 (42, 62)	55 (42, 60)	55 (42, 62)
**Ethnicity**, ***n*** **(%)**				
Caucasian	496 (89.4)	485 (87.2)	492 (88.0)	474 (85.7)
Asian	15 (2.7)	14 (2.5)	11 (2.0)	20 (3.6)
Black	6 (1.1)	8 (1.4)	13 (2.3)	13 (2.4)
Arabic	0 (0)	3 (0.5)	1 (0.2)	1 (0.2)
Hispanic	11 (2.0)	7 (1.3)	10 (1.8)	16 (2.9)
Other	27 (4.9)	39 (7.0)	32 (5.7)	29 (5.2)
Height (m)	1.68 ± 0.09	1.68 ± 0.09	1.68 ± 0.09	1.68 ± 0.10
Body weight (kg)	96.4 (83.6, 110.5)	97.3 (85.5, 111.6)	97.3 (85.5, 113.3)	95.3 (84.5, 108.6)
BMI (kg/m^2^)	33.7 (30.4, 38.5)	34.1 (30.9, 38.7)	34.2 (31.3, 39.0)	33.8 (30.3, 38.3)
Satiety (mm)	59 (50, 72)	60 (50, 75)	60 (50, 77)	59 (50, 75)
Hunger (mm)	50 (31, 65)	50 (34, 64)	50 (31, 65)	50 (30, 65)
Desire to eat (mm)	64 (50, 80)	61 (50, 79)	66 (50, 80)	62 (50, 79)
Desire to eat something	32 (10, 60)	59 (35, 79)	61 (40, 80)	55 (34, 78)
sweet (mm)				

Values represent mean ± SD for normally-distributed variables or median (25th, 75th percentiles) for non-normally-distributed variables or the number of participants (%) for categorical variables. *A total of 556 individuals were randomized to the HP-MI group, but one of them withdrew consent and requested data deletion. BMI, body mass index; HP-MI, high protein-low glycemic index diet, moderate intensity physical activity; HP-HI, high protein-low glycemic index diet, high intensity physical activity; MP-MI, moderate protein-moderate glycemic index diet, moderate intensity physical activity; MP-HI, moderate protein-moderate glycemic index diet, high intensity physical activity.

After adjusting for potential confounders, there was a significant interaction of diet × PA × time in changes of satiety (*P* = 0.038) and hunger (*P* = 0.035), whereas there was no significant diet × PA interaction (data not shown).

**Figure 2:**
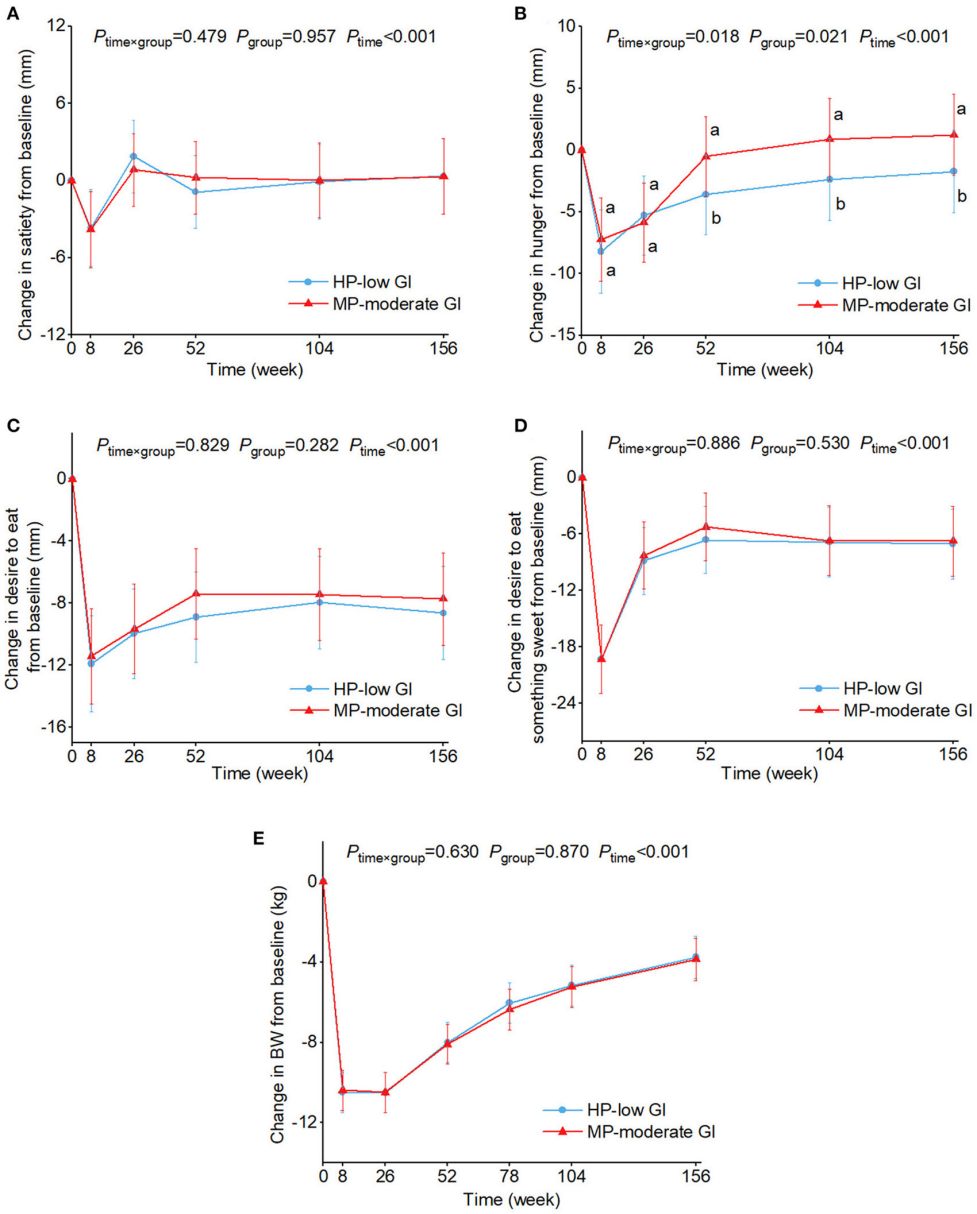
Estimated marginal means and 95% CI in changes in ratings of satiety **(A)**, hunger **(B)**, desire to eat **(C)**, and desire to eat something sweet **(D)**, and body weight **(E)** over time by two diet groups. Analyses were performed using a linear mixed model with repeated measures adjusted for age, sex, ethnicity, physical activity groups, baseline appetite ratings (0 weeks), and time as fixed effects and participant-ID and intervention center as random effects. Time×group or group×sex or group×age interaction terms were added. If the interaction was significant, *post hoc* pair-wise comparisons were conducted at each time point. Values with the different lowercase letters were significantly different, *P* < 0.05. No group×sex or group×age interaction was observed. BW, body weight; HP-low GI, high protein-low glycemic index diet; MP-moderate GI, moderate protein-moderate glycemic index diet.

Figure 2 shows the changes in appetite sensations and BW over time by the two diet groups. A significant time × diet interaction was found in changes in hunger ratings (*P* = 0.018). Hunger ratings remained significantly lower in the HP-low GI than in the MP-moderate GI group at 52, 104, and 156 weeks in the HP-low GI group than the MP-moderate GI group [changes from baseline and 95% CI: −3.6 [−6.8 to −0.3] vs. −0.5 [−3.7–2.7] mm; −2.4 [−5.7–0.9] vs. 0.9 [−2.4–4.2] mm; −1.7 [−5.0–1.6] vs. 1.2 [−2.1–4.5] mm]. There was no significant difference between the two diet groups in other appetite sensations or in BW. No group × sex or group × age interaction was observed.

**Figure 3:**
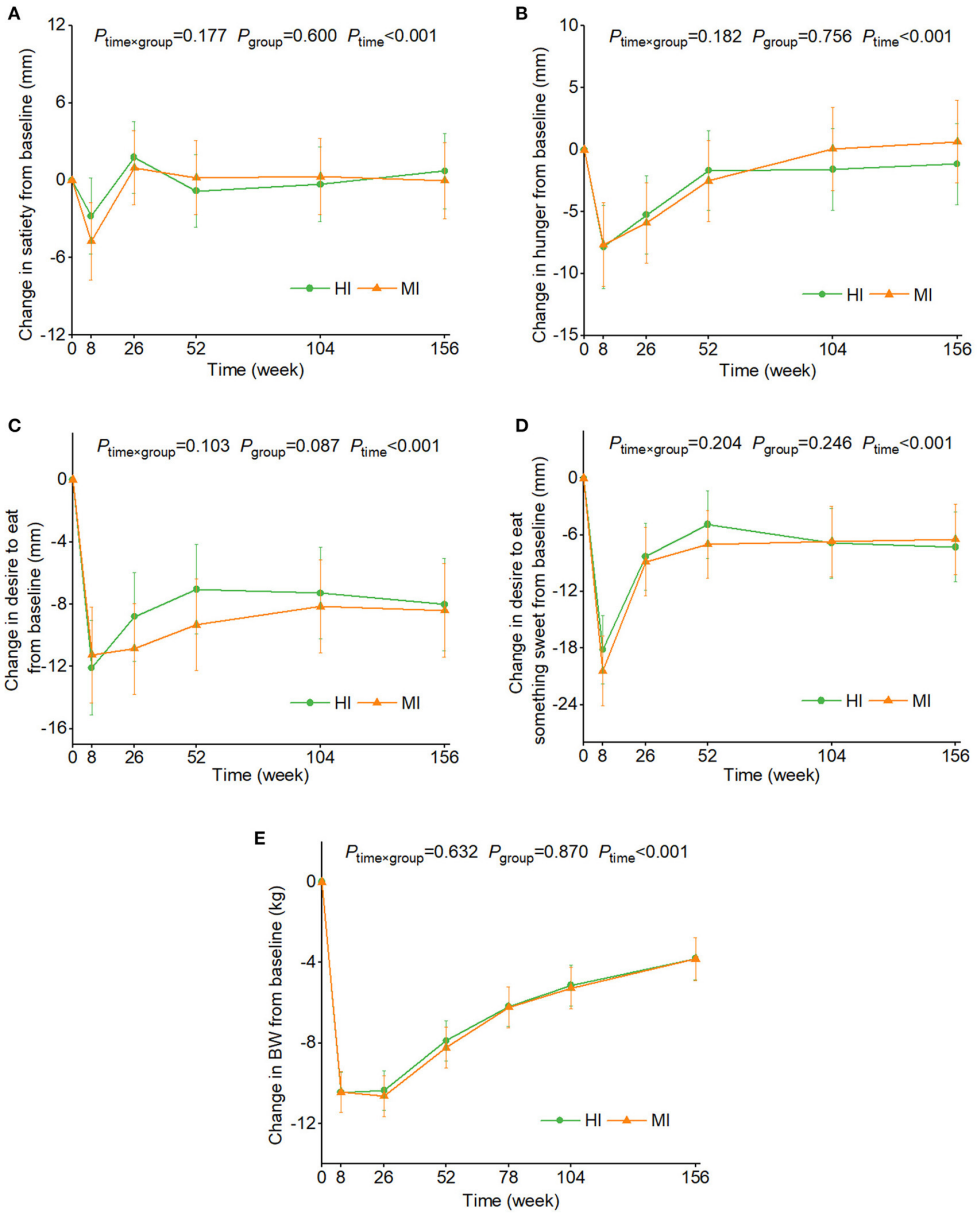
Estimated marginal means and 95% CI in changes in ratings of satiety **(A)**, hunger **(B)**, desire to eat **(C)**, and desire to eat something sweet **(D)**, and body weight **(E)** over time by two physical activity groups. Analyses were performed using a linear mixed model with repeated measures adjusted for age, sex, ethnicity, diet groups, baseline appetite ratings (0 weeks), and time as fixed effects and participant-ID and intervention center as random effects. Time × group or group × sex or group × age interaction terms were added. If the interaction was significant, *post hoc* pair-wise comparisons were conducted at each time point. Values with the different lowercase letters were significantly different, *P* < 0.05. No group × sex or group × age interaction was observed. BW, body weight; HI, high intensity physical activity; MP, moderate intensity physical activity.

Figure 3 shows the changes in appetite sensations and BW over time by two PA groups. No time × PA interaction was found and no differences were seen between the two PA groups in changes in outcomes. The sensitivity analyses revealed that changes in energy intake did not modify the results. No group × sex or group × age interaction was observed.

**Figure 4:**
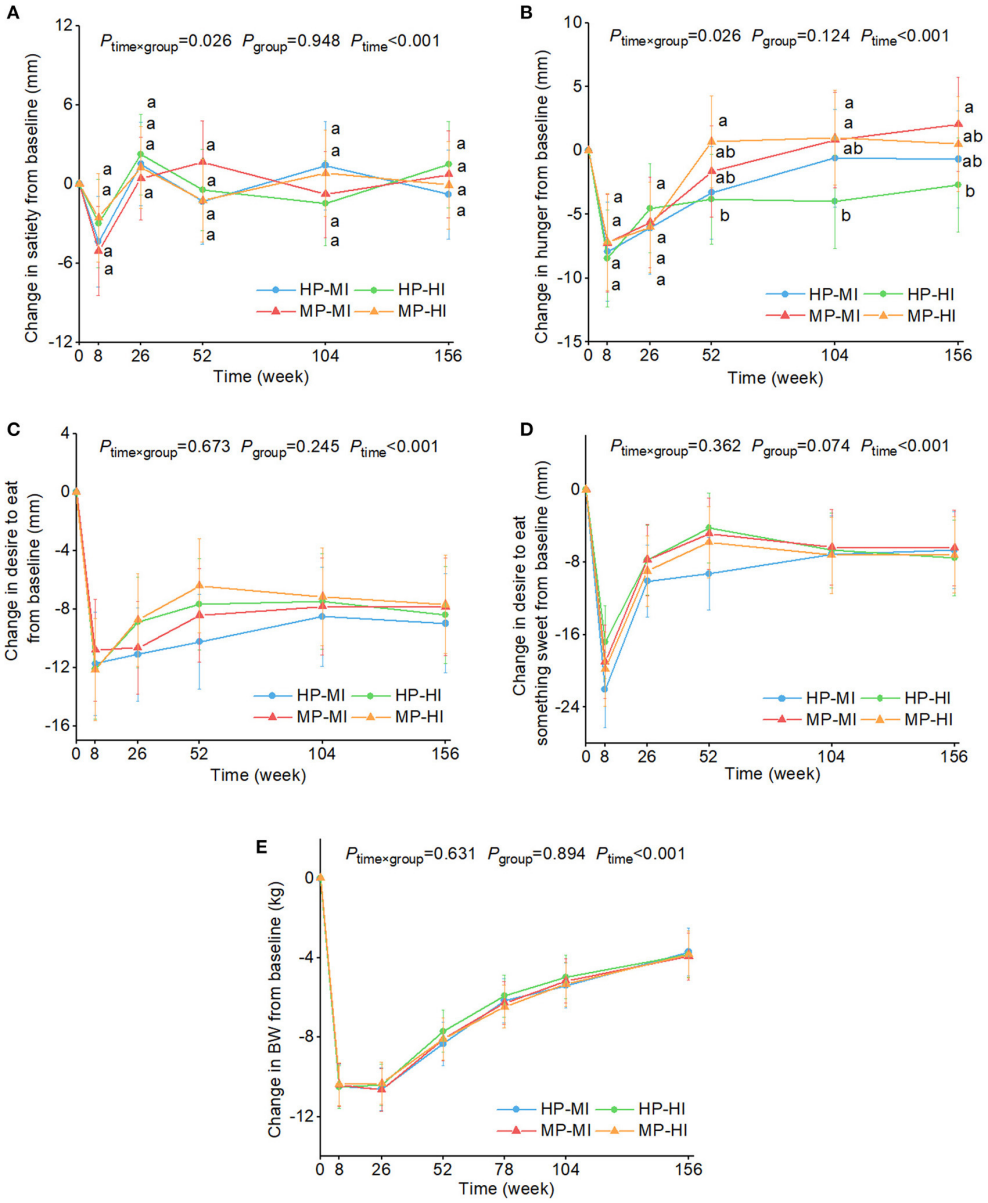
Estimated marginal means and 95% CI in changes in ratings of satiety **(A)**, hunger **(B)**, desire to eat **(C)**, and desire to eat something sweet **(D)**, and body weight **(E)** over time by four intervention groups. Analyses were performed using a linear mixed model with repeated measures adjusted for age, sex, ethnicity, baseline appetite ratings (0 weeks), and time as fixed effects and participant-ID and intervention center as random effects. Time×group or group×sex or group×age interaction terms were added. If the interaction was significant, *post hoc* multiple comparisons with Bonferroni correction were conducted at each time point. Values with the different lowercase letters were significantly different, *P* < 0.05. No group × sex or group × age interaction was observed. BW, body weight; HP-MI, high protein-low glycemic index diet, moderate intensity physical activity; HP-HI, high protein-low glycemic index diet, high intensity physical activity; MP-MI, moderate protein-moderate glycemic index diet, moderate intensity physical activity; MP-HI, moderate protein-moderate glycemic index diet, high intensity physical activity.

Figure 4 shows the changes in appetite sensation ratings and BW over time by four intervention groups. After WL at 8 weeks, ratings of satiety, hunger, desire to eat, and desire to eat something sweet, and BW were significantly lower compared with baseline (0 weeks) (*P* <0.01) in all intervention groups. Satiety and hunger, and overall appetite ratings reverted to baseline levels during the 3-years WLM, and remained at this level despite a lower BW compared with baseline. After adjusting for potential confounders, decreases in hunger ratings relative to baseline were significantly greater at 52 and 104 weeks in the HP-HI group vs. the MP-HI group [changes from baseline and 95% CI: −3.8 [−7.3 to −0.3] vs. 0.7 [−2.9–4.3] mm; −4.0 [−4.4 to −0.3] vs. 1.0 [−2.7–4.7] mm], respectively. Similarly, decreases on hunger ratings were greater at 104 and 156 weeks in the HP-HI group than the MP-MI group [−4.0 [−4.4 to −0.3] vs. 0.8 [−2.9–4.5] mm; −2.7 [−6.4–1.0] vs. 2.0 [−1.7–5.7] mm]. There were no significant differences among the four intervention groups in other appetite sensations or in BW. No group × sex or group × age interaction was observed.

Table 2 shows the correlation coefficients of relationship between change in BW and changes in appetite sensations from baseline to 156 weeks. A significant negative correlation was seen between change in satiety and change in BW from 0 to 26 weeks. Significant positive correlations were seen between vs. changes in hunger, desire to eat, and desire to eat something sweet from 0 to 26, 52, 104, and 156 weeks.

**Table 2:** Spearman correlation coefficients of relationship between change in body weight and changes in appetite sensation ratings from baseline to 156 weeks.

	**Change in body weight**			
	**0–26 weeks**	**0–52 weeks**	**0–104 weeks**	**0–156 weeks**
**Change in satiety ratings**				
0–26 weeks	−0.056^*^	-	-	-
0–52 weeks	-	−0.021	-	-
0–104 weeks	-	-	0.022	-
0–156 weeks	-	-	-	0.029
**Change in hunger ratings**				
0–26 weeks	0.183^**^	-	-	-
0–52 weeks	-	0.127^**^	-	-
0–104 weeks	-	-	0.096^**^	-
0–156 weeks	-	-	-	0.075^*^
**Change in ratings of desire to eat**				
0–26 weeks	0.140^**^	-	-	-
0–52 weeks	-	0.161^**^	-	-
0–104 weeks	-	-	0.158^**^	-
0–156 weeks	-	-	-	0.122^**^
**Change in ratings of desire to eat something sweet**				
0–26 weeks	0.160^**^	-	-	-
0–52 weeks	-	0.095^**^	-	-
0–104 weeks	-	-	0.080^**^	-
0–156 weeks	-	-	-	0.074^*^

* *P < 0.05 (2-tailed);*

** *P < 0.01 (2-tailed)*.

## Discussion

To the best of our knowledge, this is the first study to explore long-term effects and interactions of diets and PA on subjective appetite sensations after rapid LED-induced WL in a large population. Consistent with our hypothesis, we found that during the 3-years WLM phase, the HP-low GI diet was superior in suppressing hunger compared with the MP-moderate GI diet, although this did not translate to a difference in weight-regain. However, we showed significant positive correlations between changes in hunger and/or satiety and desire to eat vs. change in BW. Similarly, HP-HI suppressed hunger more than MP-HI. We did not find any difference between the two PA programs, which was contrary to our hypothesis. Surprisingly, we also showed that after 8-weeks of ≥8% weight loss using a LED (total meal replacement liquid diet), ratings of satiety, hunger, desire to eat, and desire to eat something sweet were significantly lower compared with baseline. Paradoxically, both satiety and hunger ratings decreased. This may be because, satiety and hunger were not evaluated based on acute sensations during a test meal, but based on recall of feelings during the previous week, encompassing both the fasting and postprandial state. High blood ketone levels may explain the reduction in hunger during an LED (40) but a liquid diet may not produce the same feelings of satiety as a solid meal. The DiOGenes study found a decrease in postprandial hunger and desire to eat after LED-induced rapid WL (41). Several studies have shown that WLM achieved with energy-restricted diet and/or exercise was linked to an increased drive to eat compared with baseline over 1–3 years (10–13), but most of them did not take macronutrient composition or GI or PA intensity into account (10, 12, 13). We also found that the ratings of desire to eat and desire to eat something sweet gradually increased during WLM, but did not reach the level seen in the baseline state.

In terms of macronutrient composition, we found that, compared with the MP-moderate GI group, the HP-low GI group had lower hunger ratings during WLM. This finding agrees in part with a recent systematic review which found that individuals with overweight or obesity experienced increased satiety or reduced hunger in response to HP consumption in 6 of 10 short to medium-term studies (17). However, a second meta-analysis revealed that whilst acute protein consumption may improve appetite control, the results were not consistent and heterogeneity was high in long-term trials (42). The mixed results may be partly attributed to the differences between studies, which includes the number of participants and methods of appetite assessment. In a PREVIEW sub-study (*n* = 136) conducted in Sydney, Australia, Buso et al. (43) found no difference in fasting hunger ratings between the MP-moderate GI vs. the HP-low GI groups during 3-years WLM. In that study, appetite sensations were assessed in the fasting condition, whereas in the present study, overall sensations in the previous week were recorded. In addition, the number of participants in Sydney was smaller than in the current study. Regarding methods of appetite assessment, in short-term studies with fixed meals, acute appetite was commonly determined at different time points after the meals, whereas in medium-or longer-term studies, the procedures varied. For example, Soenen et al. (44) evaluated general hunger via the Three Factor Eating Questionnaire. In a 1-year study, Cheng et al. (45) measured daily subjective appetite sensations using VAS at the end of three consecutive days for each CID and means of the three days were used (46). In the present study, appetite sensations were determined once at each CID and were based on recall of overall feelings during the prior week. Compared with measurements assessed acutely, recall of feelings may cause bias, especially when it is based on the average for a week.

Notably, we also found that participants in the HP-HI group had lower hunger ratings during WLM compared with those in the MP-HI and MP-MI groups. Without having determined other relevant outcomes, it is difficult to explain the significant differences in ratings. Further investigations would be needed if this is to be clarified.

In the present analysis, we found a significant interaction of time and four intervention groups in terms of satiety, but there were no significant differences at each CID. Westerterp-Plantenga et al. (47) found that during WLM, compared with the control group, the HP group had significantly increased satiety ratings in the fasted state before breakfast. In addition to hunger, satiety, dietary restraint, control of food-reward-related brain activation and reduced adaptive thermogenesis may have played a role in WLM of the PREVIEW study. In another PREVIEW sub-study, Drummen et al. (48) showed that HP intake reduced adaptive thermogenesis, inducing a negative energy balance compared with moderate protein intake during WLM.

Studies on the effect of GI on appetite are equivocal and controversial. A secondary observational analysis of the PREVIEW cohort found that participants in the lowest tertile of GI and glycemic load regained less weight over 3 years than the highest tertile (49). A review of short-term and medium-term studies (<1-year) found that short studies did not support an acute effect of GI on satiety, whereas the findings of longer studies were mixed (50). In another review, 12 of 18 short-term studies supported the hypothesis that low GI foods or meals lead to increased satiety or reduced hunger compared with high GI foods or meals (51). The heterogeneity in findings can probably be attributed to differences in macronutrient content, but a complicated interplay between other dietary factors can also affect both carbohydrate digestion and metabolism (50) and thereby feelings of appetite. In some studies, fiber content of the test meals was not controlled (51), although fiber intake was similar between the PREVIEW intervention groups according to the food records (31). It should be remembered, however, that we could not separate the potential effects of low GI and higher protein intake in PREVIEW, since they were both included in the HP diet. In addition, to macronutrient consumption, an individual's genotype and gut microbiota may affect appetite and food intake (16, 52). Thus, precision nutrition for appetite and weight control may be needed.

Interestingly, we found significant positive correlations between changes in ratings of hunger, desire to eat, and desire to eat something sweet vs. change in BW, when participants from the four intervention groups were merged into one group. This might partly support the generally accepted compensatory mechanism theory that any increase in drive to eat is a driver of weight regain in the long term (6–9). This result also needs to be interpreted with caution because the correlations were not strong (<0.8), and were assessed cross-sectionally. Additionally, these findings are contrary to two recent long-term studies showing no significant correlations between changes in appetite and weight regain at 1- or 2-years follow-up (12, 53). In addition to baseline BMI, study duration, and differences in procedures of appetite assessment and type of population, differences in energy intake might explain conflicting results. Unlike the referenced studies, the participants in the present study achieved partial WLM with diets varying in protein content and GI without energy limitation (i.e., *ad libitum*) rather than with energy-restricted diets.

With respect to PA, some prior studies reported no difference in appetite control between moderate-intensity continuous exercise and high-intensity interval exercise (25, 26). Taking feasibility and WLM duration into consideration, the HI program in the PREVIEW study was not designed as high-intensity *interval* exercise. Compared with moderate-PA, we found high-intensity PA did not make a difference to appetite suppression during WLM. As reported elsewhere (31), there was no difference in vigorous and moderate-to-vigorous PA between the HI and MI groups according to PREVIEW accelerometry data. The lack of PA compliance may be one of the potential reasons why we did not detect any difference in appetite sensations between the two PA groups. Indeed, all groups performed similar mean amounts of physical activity of different intensities, regardless of the physical activity intervention to which they had been assigned.

In order to encourage higher levels of compliance and behavior change, PREVIEW developed a program called PREMIT to help participants develop new diet and PA habits (33, 54) during the 3-years intervention. Data from food records and accelerometry indicated that during WLM, participants led a healthier lifestyle with higher total volume of PA (31). Nonetheless, it seems that behavioral approaches may not be sufficient for participants with overweight and obesity to achieve high intensity PA over the long term. For obese individuals in particular, HI is difficult to follow in the long-term. Previous studies found that higher BMI (>25 kg/m^2^) is linked to less power in the lower limbs and lower maximal aerobic test performance (55, 56).

The present study has several strengths. It compared two diets varying in protein content and GI according to two previous large-scale, successful intervention studies on WL (57–59). Not only the main effect but also the interaction of diet and PA was analyzed. The duration of this study was longer than in most previous trials (60), addressing a common lifelong problem of changes in appetite after diet-induced rapid WL. In addition, this study was conducted in a large overweight or obese population with a wide age span and multi-ethnicity in European countries, Australia, and New Zealand.

The present study also has limitations. The attrition rate at the end of the study approached 50%, which decreased the statistical power particularly for our main outcome (incidence of type 2 diabetes). However, for appetite scores, the current analyses have sufficient power, and attrition is not an issue (61). The level of dietary and PA compliance was poorer than planned and there was no difference between the two PA groups in PA intensity. Therefore, it remains an unanswered question if there are long-term effects of different exercise intensities on subjective appetite sensations. Further analyses on associations between PA and appetite irrespective of allocation are needed (62). Furthermore, as standard meals were not provided, all our results were based on memory recall of overall appetite sensations in the fasting and postprandial state during the previous week. Recall bias is inevitable and a combination of fasting and postprandial appetite may make some significant results undetectable. Finally, acute food intake at subsequent meals was not included in the PREVIEW protocol. As appetite sensations do not always predict individual's intake, further analyses and studies are needed.

In conclusion, a HP-low GI diet reduced feelings of hunger during WLM compared to a MP-moderate GI diet, but did not affect weight regain. In addition, the combination of HP-HI also reduced feelings of hunger compared to MP-HI. Due to insufficient difference in PA intensities between HI vs. MI, long-term differences of high vs. moderate intensity PA on appetite could not be assessed.

## Data Availability Statement

The raw data supporting the conclusions of this article will be made available by the authors, without undue reservation.

## Ethics Statement

The studies involving human participants were reviewed and approved by The PREVIEW study was approved by the Human Ethics Committees at each intervention center and was conducted in accordance with the Declaration of Helsinki and its later amendments. The patients/participants provided their written informed consent to participate in this study.

## Author Contributions

The PREVIEW project was designed by AR, JB-M, MW-P, MF, and WS. The protocol for the PREVIEW adult intervention study was written by MF, TLar, and AR. MW-P, IM, JM, SP, WS, GS, SH, NS, and MT were involved in developing the study design and diet and physical activity interventions. RZ and AR designed the analysis plan for these analyses. RZ performed the data analysis and takes responsibility for the accuracy of the data analysis. RZ drafted the manuscript with supervision from AR. AR attests that all listed authors meet authorship criteria and that no others meeting the criteria have been omitted. AR and RZ are the guarantors of this work and as such, had full access to all of the data in the study, and take responsibility for the integrity of the data. All authors contributed to the implementation of the experimental trial, contributed to analysis, interpretation of the data, contributed to critical revision of the manuscript for important intellectual content, agreed that the accuracy and integrity of the work has been appropriately investigated and resolved, and all approved the final version of the manuscript.

## Conflict of Interest

AR has received honorariums from the International Sweeteners Association and Unilever. JB-M is President and Director of the Glycemic Index Foundation, oversees of a glycemic index testing service at the University of Sydney and is a co-author of books about diet and diabetes. She is also a member of the Scientific Advisory Board of the Novo Foundation and of ZOE Global. IM was a member of the UK Government Scientific Advisory Committee on Nutrition, Treasurer of the Federation of European Nutrition Societies, Treasurer of the World Obesity Federation, member of the Mars Scientific Advisory Council, member of the Mars Europe Nutrition Advisory Board, and Scientific Adviser to the Waltham Centre for Pet Nutrition, and was also a member of the Nestle Research Scientific Advisory Board, and of the Novozymes Scientific Advisory Board, during the PREVIEW intervention. SP was the Fonterra Chair in Human Nutrition during the PREVIEW intervention. TLar is advisor for “Sense” diet program. AS owns 50% of the shares in Zuman International, a company which receives royalties for books she has written about weight management and payments for presentations at industry conferences. She has also received presentation fees and travel reimbursements from Eli Lilly and Co., the Pharmacy Guild of Australia, Novo Nordisk, the Dietitians Association of Australia, Shoalhaven Family Medical Centres, the Pharmaceutical Society of Australia, and Metagenics, and served on the Nestlé Health Science Optifast VLCD advisory board from 2016 to 2018. TLam is employed by NetUnion sarl, who contributed to the data collection process in the absence of commercial or financial conflict of interest with the study analysis. The remaining authors declare that the research was conducted in the absence of any commercial or financial relationships that could be construed as a potential conflict of interest.

## Abbreviations

BMI: body mass index
BW: body weight
GI: glycemic index
HI: high intensity physical activity
HP: higher protein
HP-HI: high protein-low glycemic index diet, moderate intensity physical activity
HP-MI: high protein-low glycemic index diet, high intensity physical activity
LED: low-energy diet
MET: metabolic equivalents of task
MI: moderate intensity physical activity
MP: moderate protein
MP-HI: moderate protein-moderate glycemic index diet, high intensity physical activity
MP-MI: moderate protein-moderate glycemic index diet, moderate intensity physical activity
OGTT: oral glucose tolerance test
PA: physical activity
VAS: visual analogue scales
WL: weight loss
WLM: weight-loss maintenance.

## References

[1] Bluher M. Obesity: global epidemiology and pathogenesis. Nat Rev Endocrinol. (2019) 15:288–98. 10.1038/s41574-019-0176-8 30814686

[2] Purcell K Sumithran P Prendergast LA Bouniu CJ Delbridge E Proietto J. The effect of rate of weight loss on long-term weight management: a randomised controlled trial. Lancet Diabetes Endocrinol. (2014) 2:954–62. 10.1016/S2213-8587(14)70200-1 25459211

[3] Aller EE Larsen TM Claus H Lindroos AK Kafatos A Pfeiffer A. Weight loss maintenance in overweight subjects on ad libitum diets with high or low protein content and glycemic index: the DIOGENES trial 12-month results. Int J Obes (Lond). (2014) 38:1511–7. 10.1038/ijo.2014.52 24675714

[4] Dombrowski SU Knittle K Avenell A Araujo-Soares V Sniehotta FF. Long term maintenance of weight loss with non-surgical interventions in obese adults: systematic review and meta-analyses of randomised controlled trials. BMJ. (2014) 348:g2646. 10.1136/bmj.g2646 25134100 PMC4020585

[5] Kraschnewski JL Boan J Esposito J Sherwood NE Lehman EB Kephart DK. Long-term weight loss maintenance in the United States. Int J Obes (Lond). (2010) 34:1644–54. 10.1038/ijo.2010.94 20479763 PMC3671378

[6] Greenway F. Physiological adaptations to weight loss and factors favouring weight regain. Int J Obes. (2015) 39:1188–96. 10.1038/ijo.2015.59 25896063 PMC4766925

[7] Polidori D Sanghvi A Seeley RJ Hall KD. How strongly does appetite counter weight loss? Quantification of the feedback control of human energy intake. Obesity. (2016) 24:2289–95. 10.1002/oby.21653 27804272 PMC5108589

[8] Muller MJ Enderle J Bosy-Westphal A. Changes in energy expenditure with weight gain and weight loss in humans. Curr Obes Rep. (2016) 5:413–23. 10.1007/s13679-016-0237-4 27739007 PMC5097076

[9] Sumithran P Proietto J. The defence of body weight: a physiological basis for weight regain after weight loss. Clin Sci (Lond). (2013) 124:231–41. 10.1042/CS20120223 23126426

[10] Sumithran P Prendergast LA Delbridge E Purcell K Shulkes A Kriketos A. Long-term persistence of hormonal adaptations to weight loss. N Engl J Med. (2011) 365:1597–604. 10.1056/NEJMoa1105816 22029981

[11] DeBenedictis JN Nymo S Ollestad KH Boyesen GA Rehfeld JF Holst JJ. Changes in the homeostatic appetite system after weight loss reflect a normalization toward a lower body weight. J Clin Endocrinol Metab. (2020) 105:e2538–46. 10.1210/clinem/dgaa202 32301981 PMC7250208

[12] Nymo S Coutinho SR Eknes PH Vestbostad I Rehfeld JF Truby H. Investigation of the long-term sustainability of changes in appetite after weight loss. Int J Obes (Lond). (2018) 42:1489–99. 10.1038/s41366-018-0119-9 29930313 PMC6113192

[13] Coutinho SR Rehfeld JF Holst JJ Kulseng B Martins C. Impact of weight loss achieved through a multidisciplinary intervention on appetite in patients with severe obesity. Am J Physiol Endocrinol Metab. (2018) 315:E91–8. 10.1152/ajpendo.00322.2017 29360396

[14] Brennan IM Luscombe-Marsh ND Seimon RV Otto B Horowitz M Wishart JM. Effects of fat, protein, and carbohydrate and protein load on appetite, plasma cholecystokinin, peptide YY, and ghrelin, and energy intake in lean and obese men. Am J Physiol Gastrointest Liver Physiol. (2012) 303:G129–40. 10.1152/ajpgi.00478.2011 22556143

[15] Hu T Yao L Reynolds K Niu T Li S Whelton P. The effects of a low-carbohydrate diet on appetite: a randomized controlled trial. Nutr Metab Cardiovasc Dis. (2016) 26:476–88. 10.1016/j.numecd.2015.11.011 26803589 PMC4873405

[16] San-Cristobal R Navas-Carretero S Martinez-Gonzalez MA Ordovas JM Martinez JA. Contribution of macronutrients to obesity: implications for precision nutrition. Nat Rev Endocrinol. (2020) 16:305–20. 10.1038/s41574-020-0346-8 32235875

[17] de Carvalho KMB Pizato N Botelho PB Dutra ES Goncalves VSS. Dietary protein and appetite sensations in individuals with overweight and obesity: a systematic review. Eur J Nutr. (2020) 59:2317–32. 10.1007/s00394-020-02321-1 32648023

[18] Samkani A Skytte MJ Thomsen MN Astrup A Deacon CF Holst JJ. Acute effects of dietary carbohydrate restriction on glycemia, lipemia and appetite regulating hormones in normal-weight to obese subjects. Nutrients. (2018) 10:91285. 10.3390/nu10091285 30213037 PMC6163561

[19] Struik NA Brinkworth GD Thompson CH Buckley JD Wittert G Luscombe-Marsh ND. Very low and higher carbohydrate diets promote differential appetite responses in adults with type 2 diabetes: a randomized trial. J Nutr. (2020) 150:800–5. 10.1093/jn/nxz344 31953540

[20] Sun F-H Li C Zhang Y-J Wong SH-S Wang L. Effect of glycemic index of breakfast on energy intake at subsequent meal among healthy people: a meta-analysis. Nutrients. (2016) 8:37. 10.3390/nu8010037 26742058 PMC4728651

[21] Krog-Mikkelsen I Sloth B Dimitrov D Tetens I Björck I Flint A. A low glycemic index diet does not affect postprandial energy metabolism but decreases postprandial insulinemia and increases fullness ratings in healthy women. J Nutr. (2011) 141:1679–84. 10.3945/jn.110.134627 21775528

[22] Donnelly JE Blair SN Jakicic JM Manore MM Rankin JW Smith BK. American College of Sports Medicine Position Stand. Appropriate physical activity intervention strategies for weight loss and prevention of weight regain for adults. Med Sci Sports Exerc. (2009) 41:459–71. 10.1249/MSS.0b013e3181949333 19127177

[23] Blundell JE Gibbons C Caudwell P Finlayson G Hopkins M. Appetite control and energy balance: impact of exercise. Obes Rev. (2015) 16 (Suppl. 1):67–76. 10.1111/obr.12257 25614205

[24] Deighton K Stensel DJ. Creating an acute energy deficit without stimulating compensatory increases in appetite: is there an optimal exercise protocol? Proc Nutr Soc. (2014) 73:352–8. 10.1017/S002966511400007X 24717417

[25] Martins C Stensvold D Finlayson G Holst J Wisloff U Kulseng B. Effect of moderate- and high-intensity acute exercise on appetite in obese individuals. Med Sci Sports Exerc. (2015) 47:40–8. 10.1249/MSS.0000000000000372 24824772

[26] Bailey DP Smith LR Chrismas BC Taylor L Stensel DJ Deighton K. Appetite and gut hormone responses to moderate-intensity continuous exercise versus high-intensity interval exercise, in normoxic and hypoxic conditions. Appetite. (2015) 89:237–45. 10.1016/j.appet.2015.02.019 25700630

[27] Matos VAF de Souza DC Browne RAV dos Santos VOA Costa EC Fayh APT. Acute effect of high-intensity interval exercise and moderate-intensity continuous exercise on appetite in overweight/obese males: a pilot study. Sport Sciences for Health. (2017) 13:403–10. 10.1007/s11332-017-0372-7

[28] Poon ET-C Sun F-H Chung AP-W Wong SH-S. Post-exercise appetite and ad libitum energy intake in response to high-intensity interval training versus moderate-or vigorous-intensity continuous training among physically inactive middle-aged adults. Nutrients. (2018) 10:1408. 10.3390/nu10101408 30279345 PMC6213307

[29] Fogelholm M Larsen TM Westerterp-Plantenga M Macdonald I Martinez JA Boyadjieva N. PREVIEW: prevention of diabetes through lifestyle intervention and population studies in Europe and around the world. Design, methods, and baseline participant description of an adult cohort enrolled into a three-year randomised clinical trial. Nutrients. (2017) 9:632. 10.3390/nu9060632 28632180 PMC5490611

[30] Christensen P Meinert Larsen T Westerterp-Plantenga M Macdonald I Martinez JA Handjiev S. Men and women respond differently to rapid weight loss: Metabolic outcomes of a multi-centre intervention study after a low-energy diet in 2500 overweight, individuals with pre-diabetes (PREVIEW). Diabetes Obes Metab. (2018) 20:2840–51. 10.1111/dom.13466 30088336 PMC6282840

[31] Raben A Vestentoft PS Brand-Miller J Jalo E Drummen M Simpson L. PREVIEW-Results from a 3-year randomised 2 x 2 factorial multinational trial investigating the role of protein, glycemic index and physical activity for prevention of type-2 diabetes. Diabetes Obes Metab. (2020) 23:324–37. 10.1111/dom.14219 33026154 PMC8120810

[32] World Health Organization. Global Recommendations on Physical Activity for Health. Available online at: https://www.who.int/dietphysicalactivity/global-PA-recs-2010.pdf (accessed January 17, 2021).

[33] Kahlert D Unyi-Reicherz A Stratton G Meinert Larsen T Fogelholm M Raben A. PREVIEW behavior modification intervention toolbox (PREMIT): a study protocol for a psychological element of a multicenter project. Front Psychol. (2016) 7:1136. 10.3389/fpsyg.2016.01136 27559319 PMC4978707

[34] American Diabetes Association. 2. classification and diagnosis of diabetes. Diabetes Care. (2017) 40:S11–S24. 10.2337/dc17-S005 27979889

[35] Silventoinen K Pankow J Lindstrom J Jousilahti P Hu G Tuomilehto J. The validity of the Finnish Diabetes Risk Score for the prediction of the incidence of coronary heart disease and stroke, and total mortality. Eur J Cardiovasc Prev Rehabil. (2005) 12:451–8. 10.1097/01.hjr.0000174793.31812.21 16210931

[36] Womble LG Wadden TA Chandler JM Martin AR. Agreement between weekly vs. daily assessment of appetite. Appetite. (2003) 40:131–5. 10.1016/S0195-6663(02)00170-8 12781162

[37] Martin CK Rosenbaum D Han H Geiselman PJ Wyatt HR Hill JO. Change in food cravings, food preferences, and appetite during a low-carbohydrate and low-fat diet. Obesity. (2011) 19:1963–70. 10.1038/oby.2011.62 21494226 PMC3139783

[38] Stubbs RJ Hughes DA Johnstone AM Rowley E Reid C Elia M. The use of visual analogue scales to assess motivation to eat in human subjects: a review of their reliability and validity with an evaluation of new hand-held computerized systems for temporal tracking of appetite ratings. Br J Nutr. (2000) 84:405–15. 10.1017/S0007114500001719 11103211

[39] Kahan BC Morris TP. Reporting and analysis of trials using stratified randomisation in leading medical journals: review and reanalysis. BMJ. (2012) 345:e5840. 10.1136/bmj.e5840 22983531 PMC3444136

[40] Colles SL Dixon JB Marks P Strauss BJ O'Brien PE. Preoperative weight loss with a very-low-energy diet: quantitation of changes in liver and abdominal fat by serial imaging. Am J Clin Nutr. (2006) 84:304–11. 10.1093/ajcn/84.1.304 16895876

[41] Andriessen C Christensen P Vestergaard Nielsen L Ritz C Astrup A Meinert Larsen T. Weight loss decreases self-reported appetite and alters food preferences in overweight and obese adults: observational data from the DiOGenes study. Appetite. (2018) 125:314–22. 10.1016/j.appet.2018.02.016 29471068

[42] Kohanmoo A Faghih S Akhlaghi M. Effect of short- and long-term protein consumption on appetite and appetite-regulating gastrointestinal hormones, a systematic review and meta-analysis of randomized controlled trials. Physiol Behav. (2020) 226:113123. 10.1016/j.physbeh.2020.113123 32768415

[43] Buso ME Mcclintock RV McClintock S Muirhead R Atkinson FS Brodie S. Can a higher protein/low glycemic index versus a conventional diet attenuate changes in appetite and gut hormones following weight loss? A 3-year PREVIEW sub-study. Front Nutr. (2021) 8:640538. 10.3389/fnut.2021.640538 33829034 PMC8019730

[44] Soenen S Martens EA Hochstenbach-Waelen A Lemmens SG Westerterp-Plantenga MS. Normal protein intake is required for body weight loss and weight maintenance, and elevated protein intake for additional preservation of resting energy expenditure and fat free mass. J Nutr. (2013) 143:591–6. 10.3945/jn.112.167593 23446962

[45] Cheng HL Griffin H Claes BE Petocz P Steinbeck K Rooney K. Influence of dietary macronutrient composition on eating behaviour and self-perception in young women undergoing weight management. Eat Weight Disord. (2014) 19:241–7. 10.1007/s40519-014-0110-y 24609724

[46] Kjølbæk L Sørensen LB Søndertoft NB Rasmussen CK Lorenzen JK Serena A. Protein supplements after weight loss do not improve weight maintenance compared with recommended dietary protein intake despite beneficial effects on appetite sensation and energy expenditure: a randomized, controlled, double-blinded trial. Am J Clin Nutr. (2017) 106:684–97. 10.3945/ajcn.115.129528 28679554

[47] Westerterp-Plantenga MS Lejeune MP Nijs I van Ooijen M Kovacs EM. High protein intake sustains weight maintenance after body weight loss in humans. Int J Obes Relat Metab Disord. (2004) 28:57–64. 10.1038/sj.ijo.0802461 14710168

[48] Drummen M Tischmann L Gatta-Cherifi B Fogelholm M Raben A Adam TC. High compared with moderate protein intake reduces adaptive thermogenesis and induces a negative energy balance during long-term weight-loss maintenance in participants with prediabetes in the postobese state: a PREVIEW study. J Nutr. (2020) 150:458–63. 10.1093/jn/nxz281 31754687 PMC7056617

[49] Zhu R Larsen TM Fogelholm M Poppitt S Vestentoft PS Silvestre MP. Dose-dependent associations of dietary glycemic index, glycemic load and fiber with 3-year weight-loss maintenance and glycemic status in a high-risk population: a secondary analysis of the PREVIEW diabetes prevention study. Diabetes Care. 44 (in press). 10.2337/dc20-3092 PMC8323188 34045241

[50] Vega-López S Venn BJ Slavin JL. Relevance of the glycemic index and glycemic load for body weight, diabetes, and cardiovascular disease. Nutrients. (2018) 10:1361. 10.3390/nu10101361 30249012 PMC6213615

[51] Bornet FR Jardy-Gennetier AE Jacquet N Stowell J. Glycaemic response to foods: impact on satiety and long-term weight regulation. Appetite. (2007) 49:535–53. 10.1016/j.appet.2007.04.006 17610996

[52] Cuevas-Sierra A Ramos-Lopez O Riezu-Boj JI Milagro FI Martinez JA. Diet, gut microbiota, and obesity: links with host genetics and epigenetics and potential applications. Adv Nutr. (2019) 10:S17–S30. 10.1093/advances/nmy078 30721960 PMC6363528

[53] Nymo S Coutinho SR Rehfeld JF Truby H Kulseng B Martins C. Physiological predictors of weight regain at 1-year follow-up in weight-reduced adults with obesity. Obesity. (2019) 27:925–31. 10.1002/oby.22476 31004405 PMC6593985

[54] Huttunen-Lenz M Hansen S Christensen P Meinert Larsen T Sando-Pedersen F Drummen M. PREVIEW study-influence of a behavior modification intervention (PREMIT) in over 2300 people with pre-diabetes: intention, self-efficacy and outcome expectancies during the early phase of a lifestyle intervention. Psychol Res Behav Manag. (2018) 11:383–94. 10.2147/PRBM.S160355 30254498 PMC6143124

[55] Nikolaidis PT. Association between body mass index, body fat per cent and muscle power output in soccer players. Cent Eur J Med. (2012) 7:783–9. 10.2478/s11536-012-0057-1

[56] Nikolaidis P. Weight status and physical fitness in female soccer players: is there an optimal BMI? Sport Sci Health. (2014) 10:41–8. 10.1007/s11332-014-0172-2

[57] Larsen TM Dalskov SM van Baak M Jebb SA Papadaki A Pfeiffer AF. Diets with high or low protein content and glycemic index for weight-loss maintenance. N Engl J Med. (2010) 363:2102–13. 10.1056/NEJMoa1007137 21105792 PMC3359496

[58] Eriksson J Lindstrom J Valle T Aunola S Hamalainen H Ilanne-Parikka P. Prevention of Type II diabetes in subjects with impaired glucose tolerance: the Diabetes Prevention Study (DPS) in Finland. Study design and 1-year interim report on the feasibility of the lifestyle intervention programme. Diabetologia. (1999) 42:793–801. 10.1007/s001250051229 10440120

[59] Physical Activity Guidelines for Americans. Available online at: http://www.health.gov/paguidelines/guidelines/ (accessed 23 October 2020).

[60] Fogelholm M Kukkonen-Harjula K. Does physical activity prevent weight gain–a systematic review. Obes Rev. (2000) 1:95–111. 10.1046/j.1467-789x.2000.00016.x 12119991

[61] Flint A Raben A Blundell J Astrup A. Reproducibility, power and validity of visual analogue scales in assessment of appetite sensations in single test meal studies. Int J Obes (Lond). (2000) 24:38–48. 10.1038/sj.ijo.0801083 10702749

[62] Swindell N Rees P Fogelholm M Drummen M MacDonald I Martinez JA. Compositional analysis of the associations between 24-h movement behaviours and cardio-metabolic risk factors in overweight and obese adults with pre-diabetes from the PREVIEW study: cross-sectional baseline analysis. Int J Behav Nutr Phys Act. (2020) 17:29. 10.1186/s12966-020-00936-5 32131847 PMC7055067

