# Corrigendum: A High-Protein, Low Glycemic Index Diet Suppresses Hunger but Not Weight Regain After Weight Loss: Results From a Large, 3-Years Randomized Trial (PREVIEW)

**DOI:** 10.3389/fnut.2021.736531

**Published:** 2021-07-23

**Authors:** Ruixin Zhu, Mikael Fogelholm, Thomas M. Larsen, Sally D. Poppitt, Marta P. Silvestre, Pia S. Vestentoft, Elli Jalo, Santiago Navas-Carretero, Maija Huttunen-Lenz, Moira A. Taylor, Gareth Stratton, Nils Swindell, Niina E. Kaartinen, Tony Lam, Teodora Handjieva-Darlenska, Svetoslav Handjiev, Wolfgang Schlicht, J. Alfredo Martinez, Radhika V. Seimon, Amanda Sainsbury, Ian A. Macdonald, Margriet S. Westerterp-Plantenga, Jennie Brand-Miller, Anne Raben

**Affiliations:** ^1^Department of Nutrition, Exercise and Sports, Faculty of Science, University of Copenhagen, Copenhagen, Denmark; ^2^Department of Food and Nutrition, University of Helsinki, Helsinki, Finland; ^3^Human Nutrition Unit, School of Biological Sciences, Department of Medicine, University of Auckland, Auckland, New Zealand; ^4^Center for Health Technology Services Research, NOVA Medical School, Universidade Nova de Lisboa, Lisbon, Portugal; ^5^Centre for Nutrition Research, University of Navarra, Pamplona, Spain; ^6^Centro de Investigacion Biomedica en Red Area de Fisiologia de la Obesidad y la Nutricion (CIBEROBN), Madrid, Spain; ^7^IdisNA Instituto for Health Research, Pamplona, Spain; ^8^Institute for Nursing Science, University of Education Schwäbisch Gmünd, Schwäbisch Gmünd, Germany; ^9^Division of Physiology, Pharmacology and Neuroscience, School of Life Sciences, Queen's Medical Centre, Nottingham, United Kingdom; ^10^National Institute for Health Research (NIHR) Nottingham Biomedical Research Centre, Nottingham, United Kingdom; ^11^Applied Sports, Technology, Exercise and Medicine (A-STEM) Research Centre, Swansea University, Swansea, United Kingdom; ^12^Department of Public Health and Welfare, Finnish Institute for Health and Welfare, Helsinki, Finland; ^13^NetUnion sarl, Lausanne, Switzerland; ^14^Department of Pharmacology and Toxicology, Medical University of Sofia, Sofia, Bulgaria; ^15^Exercise and Health Sciences, University of Stuttgart, Stuttgart, Germany; ^16^Department of Nutrition and Physiology, University of Navarra, Pamplona, Spain; ^17^Precision Nutrition and Cardiometabolic Health Program, IMDEA (Madrid Institute for Advanced Studies)-Food Institute, CEI UAM + CSIC (Campus de Excelencia Internacional, Universidad Autónoma de Madrid + Consejo Superior de Investigaciones Científicas), Madrid, Spain; ^18^The Boden Collaboration for Obesity, Nutrition, Exercise, and Eating Disorders, Faculty of Medicine and Health, Charles Perkins Centre, The University of Sydney, Camperdown, NSW, Australia; ^19^School of Human Sciences (Exercise and Sports Science), Faculty of Science, The University of Western Australia, Crawley, WA, Australia; ^20^Division of Physiology, Pharmacology and Neuroscience, School of Life Sciences, Queen's Medical Centre, MRC/ARUK Centre for Musculoskeletal Ageing Research, ARUK Centre for Sport, Exercise and Osteoarthritis, National Institute for Health Research (NIHR) Nottingham Biomedical Research Centre, Nottingham, United Kingdom; ^21^Department of Nutrition and Movement Sciences, NUTRIM, School of Nutrition and Translational Research in Metabolism, Maastricht University, Maastricht, Netherlands; ^22^School of Life and Environmental Sciences and Charles Perkins Centre, The University of Sydney, Sydney, NSW, Australia; ^23^Steno Diabetes Center Copenhagen, Gentofte, Denmark

**Keywords:** obesity, pre-diabetes, satiety, desire to eat, low-energy diet, weight-loss maintenance

In the published article, there was an error in affiliations 5, 6 and 7. Instead of “5 Department of Nutrition, University of Navarra, Pamplona, Spain, 6 CIBERobn, Instituto de Salud Carlos III, Madrid, Spain, 7 Precision Nutrition Program, IMDEA Food, Campus de Excelencia Internacional, Universidad Autónoma de Madrid + Consejo Superior de Investigaciones Científicas, Madrid, Spain”, it should be “5 Centre for Nutrition Research, University of Navarra, Pamplona, Spain, 6 Centro de Investigacion Biomedica en Red Area de Fisiologia de la Obesidad y la Nutricion (CIBEROBN), Madrid, Spain, 7 IdisNA Instituto for Health Research, Pamplona, Spain”.

In the published article, there was an error regarding the affiliations for J. Alfredo Martinez. As well as having affiliations 6 and 7, they should also have “16 Department of Nutrition and Physiology, University of Navarra, Pamplona, Spain” and “17 Precision Nutrition and Cardiometabolic Health Program, IMDEA (Madrid Institute for Advanced Studies)-Food Institute, CEI UAM + CSIC (Campus de Excelencia Internacional, Universidad Autónoma de Madrid + Consejo Superior de Investigaciones Científicas), Madrid, Spain”

In the original article, there was an error. In the Conflict of Interest statement, “TLar is advisor for “Sense” diet program AS owns 50% of the shares in Zuman International” should be “TLar is advisor for “Sense” diet program. AS owns 50% of the shares in Zuman International”

In the original article, there was an error. In the Conflict of Interest statement, “JB-M was President and Director of the Glycemic Index Foundation” should be “JB-M is President and Director of the Glycemic Index Foundation”.

The authors apologize for these errors and state that these do not change the scientific conclusions of the article in any way. The original article has been updated.

## Publisher's Note

All claims expressed in this article are solely those of the authors and do not necessarily represent those of their affiliated organizations, or those of the publisher, the editors and the reviewers. Any product that may be evaluated in this article, or claim that may be made by its manufacturer, is not guaranteed or endorsed by the publisher.

